# Rare Manifestation of Pancreaticobiliary Cancer With Extrahepatic Lesions

**DOI:** 10.7759/cureus.43276

**Published:** 2023-08-10

**Authors:** Ruochen Ying, Anmol Mittal, Kaveh Hajifathalian

**Affiliations:** 1 Department of Medicine, Rutgers University, New Jersey Medical School, Newark, USA; 2 Department of Gastroenterology and Hepatology, Rutgers University, New Jersey Medical School, Newark, USA

**Keywords:** liver, lung, rare, metastasis, bone, biliary, pancreatic, carcinoma, pancreaticobiliary

## Abstract

Pancreaticobiliary cancers are uncommon neoplasms, frequently diagnosed at an advanced stage with metastasis to the liver, lung, or peritoneum. Here, we report an extraordinary case of a patient presenting with both hepatic and extrahepatic lesions, including bone involvement, but without discernible disease in the biliary system or pancreas. Eventual pathological evidence supports the origin of primary pancreaticobiliary carcinoma. This case report aims to stimulate discourse regarding the consideration of pancreaticobiliary cancers as a potential cause of extrahepatic metastatic lesions. Increased awareness of such atypical presentations is crucial for early diagnosis and optimal patient management.

## Introduction

Pancreaticobiliary cancers are uncommon in the United States. Pancreatic cancers are estimated to account for about 3% of new cases in 2022, and biliary cancers even less [1-3]. About 45%-55% of pancreatic cancers are stage IV on diagnosis [4]. Metastatic sites for pancreatic cancers include the peritoneum/omentum, liver, lungs, and distant lymph nodes [5]. Similarly, biliary cancers, of which gallbladder cancer is the most common, usually metastasize to the liver, adjacent structures, and lymph nodes [6]. Extrahepatic metastases are rare but include bony and skeletal sites like the spine [7,8]. A retrospective survey to explore the natural history of such cancer demonstrated a correlation between bony lesions on initial presentation and poor prognosis [8]. We present a rare case of a patient with pancreaticobiliary cancer with metastatic lesions to the lungs, liver, and spine at presentation.

## Case presentation

An 80-year-old male with a medical history of hypertension, diabetes, tobacco use disorder, alcohol use disorder, and hepatitis C infection with sustained virologic response presented with three weeks of worsening generalized weakness, fatigue, lower back pain that awakened him at night, abdominal pain, constipation, urinary incontinence, and unintentional weight loss. He denied any fevers, chills, night sweats, cough, chest pain, palpitations, shortness of breath, nausea, vomiting, diarrhea, melena, or bright red blood per rectum. He previously smoked cigarettes before quitting several years ago. The patient was uncertain about the pack years but reported many years of tobacco use. The patient had family members with cancer of an unknown type.

Initial vitals were a temperature of 97°F, a pulse rate of 116 beats per minute, and a blood pressure of 96/63 mmHg. He had leukocytosis, hyperkalemia, hypercalcemia, acute kidney injury, alkaline phosphatemia, elevated liver enzymes with a mildly elevated direct bilirubin of 0.4 mg/dl, and a protein gap of 5.4 g/dl (Table 1).

**Table 1:** The patient's initial chemistry and complete blood count results on presentation

Component and Results	Reference Range
Blood Urea Nitrogen: 68	8 - 23 mg/dl
Creatinine: 2.0	0.7 - 1.2 mg/dL
Sodium: 139	133 - 145 meq/l
Potassium: 5.8	3.5 - 4.8 meq/l
Chloride: 103	97 - 110 meq/l
Bicarbonate: 23	23 - 30 meq/l
Calcium: 13.1	8.8 - 10.2 mg/dl
Phosphorus: 3.8	2.5 - 4.5 mg/dl
Total Protein: 8.7	6.0 - 8.3 gm/dl
Albumin: 3.3	3.5 - 5.2 gm/dL
Alkaline Phosphatase: 509	40 - 130 u/l
Bilirubin Direct: 0.4	<=0.3 mg/dl
Bilirubin Total: 0.8	<=1.0 mg/dl
Aspartate Aminotransferase: 129	0 - 40 U/L
Alanine Aminotransferase: 104	0 - 41 U/L
Lipase: 53	13 - 60 u/l
Magnesium: 2.7	1.6 - 2.5 mg/dL
Lactic Acid: 2.6	0.5 - 2.2 mmol/l
White Blood Cells: 14.9	4.0 - 11.0 x 10*3/µL
Red Blood Cells: 6.51	4.70 - 6.10 x 10*6/µL
Hemoglobin: 17.1	14.0 - 18.0 g/dL
Hematocrit: 52.1	42.0 - 54.0 %
Mean Corpuscular Volume: 80.1	80.0 - 99.0 fl
Mean Corpuscular Hemoglobin: 26.3	27.0 - 33.0 pg
Mean Corpuscular Hemoglobin Concentration: 32.8	32.0 - 36.0 g/dL
Red Cell Distribution Width: 14.1	11.0 - 15.0 %
Platelets: 162	150 - 450 x 10*3/µL
Neutrophil %: 83.9	35.0 - 80.0 %
Lymphocyte %: 8.1	20.0 - 50.0 %
Monocyte %: 7.6	2.0 - 12.0 %
Eosinophil %: 0.2	0.0 - 7.0 %
Basophil %: 0.2	0.0 - 2.0 %
Neutrophils (Absolute): 12.5	x 10*3/µL
Lymphocyte (Absolute): 1.2	x 10*3/µL
Monocyte (Absolute): 1.1	x 10*3/µL
Eosinophil (Absolute): 0.0	x 10*3/µL
Basophil (Absolute): 0.0	x 10*3/µL

**Figure 1:**
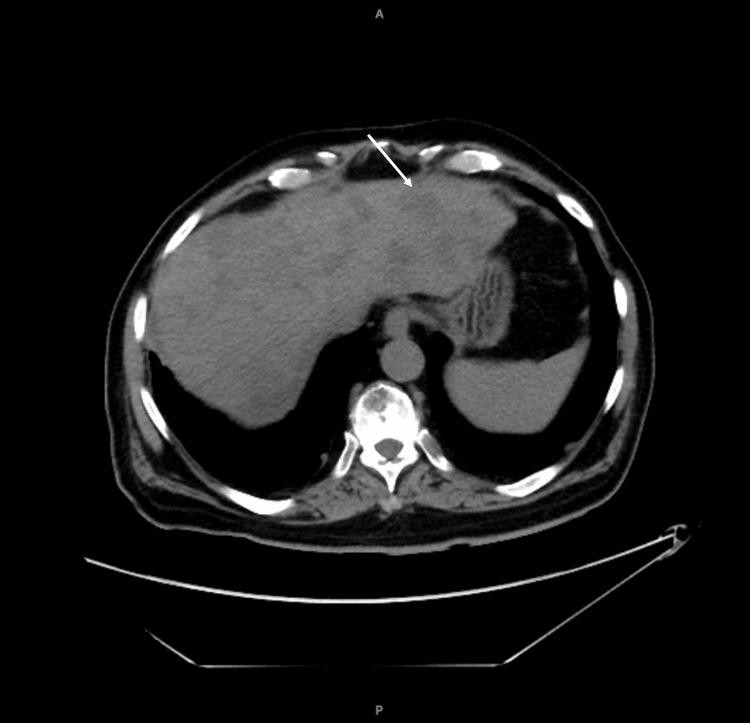
Computed tomography (CT) scans without contrast show numerous hepatic lesions

**Figure 2:**
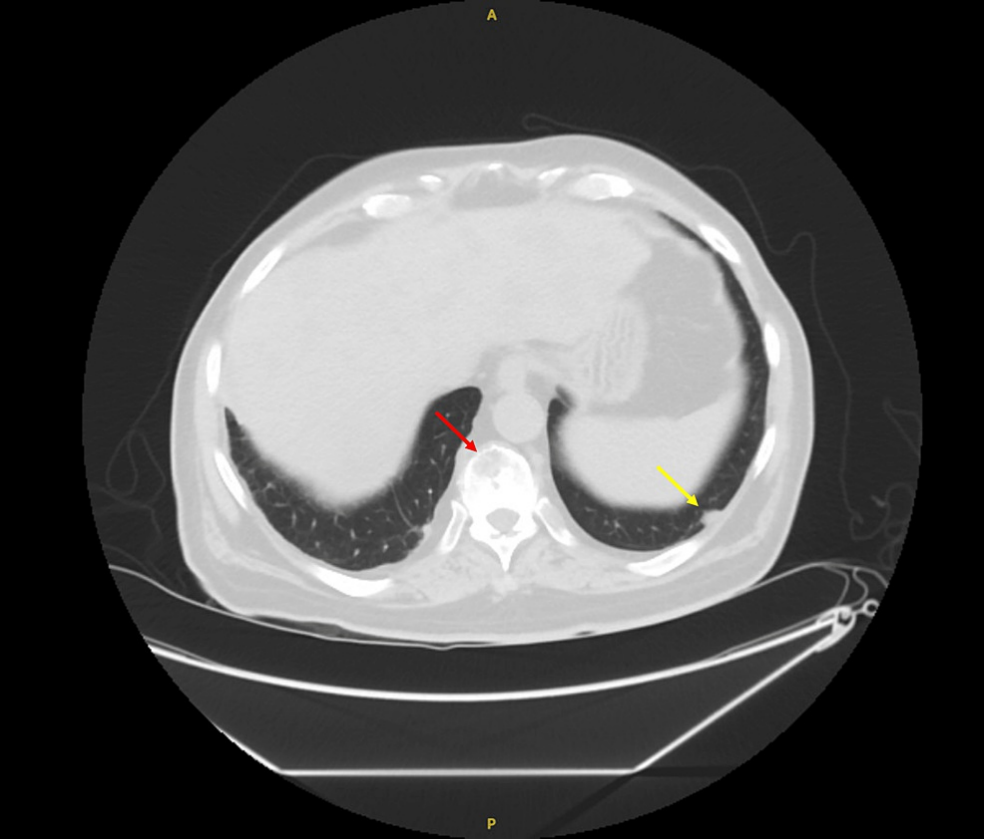
The CT scan shows one of the multiple lung lesions (yellow arrow) and one of the multiple lytic bony lesions (red arrow).

The chest X-ray showed right perihilar consolidation. An ultrasound of the abdomen demonstrated cirrhosis, portal hypertension, and multiple masses. There was also cholecystolithiasis without specific sonographic features of acute cholecystitis. Computed tomography (CT) scans without contrast later showed innumerable hypoattenuating hepatic lesions (Figure 1), with multiple enlarged and heterogeneous porta hepatis lymph nodes, numerous pulmonary nodules, and vertebral body lytic lesions compatible with metastatic disease (Figure 2).

The exact primary site could not be identified at the time. The hepatitis C viral load was undetectable. His serum light chains show an elevated kappa-to-lambda ratio of 2.02. Carbohydrate antigen 19-9 (CA 19-9) was 2717 U/mL (reference range: 0-35 U/mL), and cancer antigen 125 was 142 U/mL (reference range: 0-35 U/mL). His prostate-specific antigen was 5.8 ng/mL (reference range: 0-4 ng/mL). Serum protein electrophoresis was consistent with a monoclonal gammopathy, likely of the immunoglobulin M (IgM) component. Magnetic resonance imaging (MRI) of the thoracic spine showed bony metastasis involving the T11 vertebral body and the left pedicle.

Sadly, the patient’s condition, including his mental status, continued to deteriorate. The CT imaging of the head resulted in negative results for acute lesions or strokes. A biopsy of a liver lesion was obtained by interventional radiology. Although treatment for hypercalcemia marginally improved his mental status, the patient never returned to baseline. After lengthy goals of care discussions and per the patient’s wishes, the family chose hospice.

**Figure 3:**
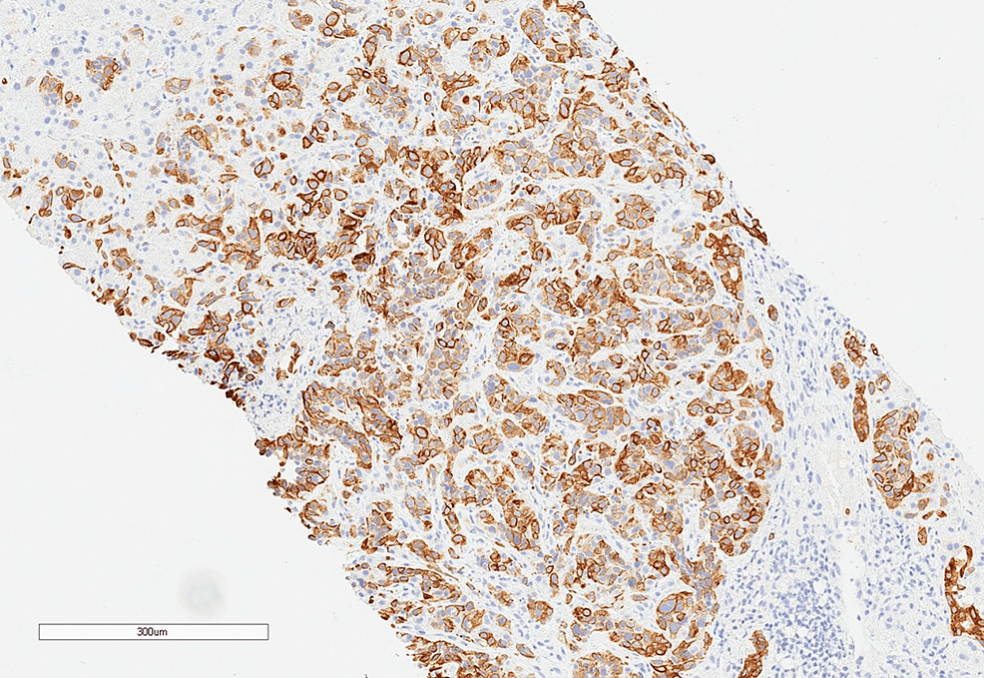
The immunohistochemistry stain (10x magnification) for CK7 is positive. CK: cytokeratin

**Figure 4:**
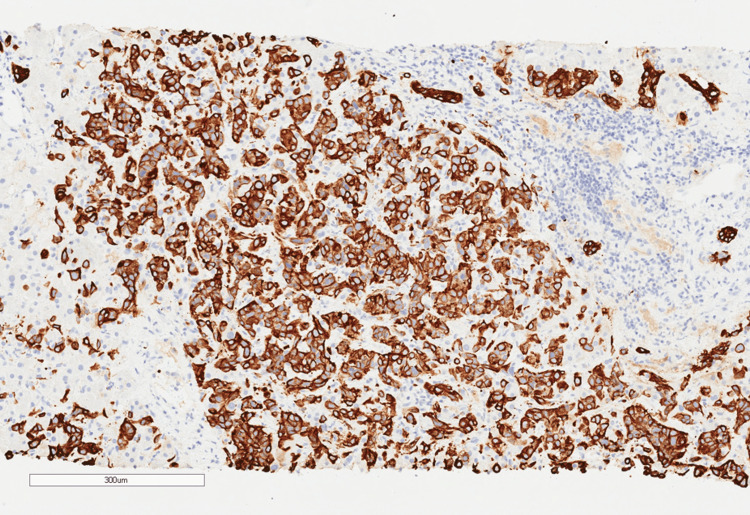
The immunohistochemistry stain (10x magnification) for CK19 is strongly positive. CK: cytokeratin

A few days later, pathology results of the liver biopsy demonstrated the cells to be positive for cytokeratin AE1/AE3, cytokeratin 7 (CK7) (Figure 3), and cytokeratin 19 (CK19) (Figure 4).

**Figure 5:**
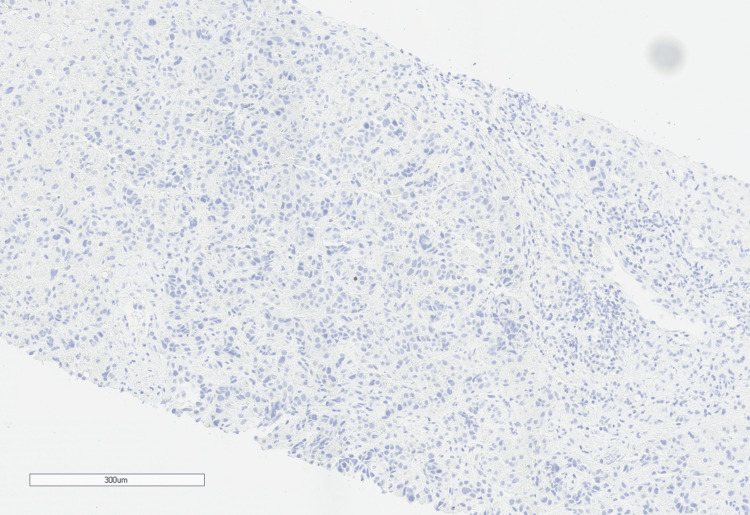
The immunohistochemistry stain (10x magnification) for TTF-1 is negative. TTF-1: thyroid transcription factor 1

Cytokeratin 20 (CK20) was focally positive. Immunostain HepPar1 and Arginase-1 had scattered positivity. Immunostains for thyroid transcription factor 1 (TTF1) (Figure 5) and NKX3.1 were negative.

His liver biopsy morphologic and immunohistochemical profile was most compatible with pancreaticobiliary carcinoma. Specifically, CK7 and CK19 positivity support a cholangiocarcinoma diagnosis. HepPar1 can be positive for either hepatocellular carcinoma (HCC) or pancreaticobiliary cancer. Cytokeratin 7 (CK7) and CK20 are also typically positive in pancreatic adenocarcinoma. Thyroid transcription factor 1 is typically positive in HCC but only positive in about 10% of cholangiocarcinomas; so, this being negative further supports pancreaticobiliary origin.

## Discussion

In summary, the patient was an elderly man who presented with generalized weakness, fatigue, weight loss, and numerous lesions concerning metastatic carcinoma of unknown origin. Initially, we thought the primary cancer must be either liver or lung, as each commonly metastasizes to the other site, and both share sites of metastasis, including bone and adrenal glands. Lung cancers also produce lytic bone lesions [9, 10]. The CA 19-9 test supported possible pancreaticobiliary cancer, but we considered it lower on our differential as there were no lesions seen in the pancreas or biliary tree on CT imaging. Our team had considered further studies, including an MRI of the abdomen, but the family decided not to pursue them and favored palliation.

A literature review found pancreaticobiliary cancer to be rare. Although most cases of pancreaticobiliary cancer present with metastatic disease, especially to the liver and lungs, the spread of the disease to the bone is less frequent [7,8]. A retrospective analysis of cancer characteristics done by Santini et al. in patients who had died of biliary cancer with bone metastases showed that 68.6% of these patients had metastatic disease somewhere in the body at the time of initial diagnosis, and only 35% had lesions to the bone on presentation [8]. Similarly, Wang et al. saw that the distribution of bone metastases in extrahepatic bile-duct cancers was one of the fewer sites as compared to liver and distant lymph nodes [7]. They even looked at the combined metastatic patterns, and it turns out lung metastasis is a more common site for bi-site combined metastasis. The synchronous metastasis of the liver and lung is more frequent than any other co-metastasis combined with the liver. This was also seen in our patient, who had numerous lesions in his liver and lungs. Interestingly, the study also found bone metastasis preferentially co-metastasize with brain and lung, and though our patient did not have a brain lesion on CT, he did have a rapidly declining mental status. These patterns suggest that pancreaticobiliary cancer should still be high on the differential with lesions in the liver and lung without a conspicuous primary mass.

Wang et al. also found bone combined lung metastasis had a decreased survival rate compared to their respective separate single metastasis [7]. Santini et al. found that the median overall survival (mOS) of patients with bone metastases from biliary cancer was only six months, compared to 16.5 months for patients with biliary cancer. Interestingly, patients who were treated with bisphosphonates had a significantly higher mOS than those who were not, which is evidence supportive of a poor prognosis for patients with bone metastases [8]. Though these results cannot explain our patient’s fast decline, they highlight the importance of identifying survival rates of different metastatic patterns to give guidance to clinicians in end-of-life conversations with patients and families.

Due to new discoveries in oncologic drivers, recent years have seen large advances in chemotherapy and biologics targeting tumor biomarkers [11-14]. Additionally, pancreaticobiliary cancers that present as advanced diseases have an unresectable tumor burden, so the focus of treatment has now increasingly involved non-surgical interventions. Some notable therapies include the combination of gemcitabine and cisplatin or biologics like ivosidenib (mutant isocitrate dehydrogenase-1 inhibitor) or bevacizumab (anti-vascular endothelial growth factor A) [6, 12, 15]. Patients treated with gemcitabine and cisplatin had higher mOS compared to those treated with gemcitabine monotherapy [6, 11, 16]. Patients, like the one in this case report, may also benefit from palliative approaches, and some serious considerations have been given to procedures like biliary drainage with endoscopically or percutaneously placed stents, radiofrequency ablation, radioembolization, photodynamic therapy, proton beam therapy, hepatic arterial infusion chemotherapy, transarterial chemoembolization, and stereotactic radiotherapy [6, 11, 15]. All in all, the treatment landscape for pancreaticobiliary cancers is evolving, and the future may hold more promising options for patients.

## Conclusions

The occurrence of pancreaticobiliary carcinoma is uncommon, but the patient usually presents with metastatic lesions on initial diagnosis, given the inconspicuous nature of the disease. The pattern of metastasis of pancreaticobiliary carcinoma commonly includes the liver and lung, but in this patient, there were also metastases to the bone, without obvious evidence of intrabiliary or intrapancreatic disease. This case serves as a reminder to keep a higher clinical suspicion for pancreaticobiliary carcinoma as a differential in patients with extrahepatic lesions. Additionally, the patient’s rapid mental decline highlights the importance of identifying associations between metastatic patterns and prognosis to help guide the goals of care conversations.
